# Interstitial Ectopic Pregnancy: A Rare Diagnosis

**DOI:** 10.7759/cureus.43107

**Published:** 2023-08-08

**Authors:** Rita Palma, Catarina Silva, Ana Tomé, Elisa Pereira, Ana Filipa Regalo

**Affiliations:** 1 Obstetrics and Gynecology, Hospital Garcia de Orta, Almada, PRT; 2 Gynecologic Oncology, Hospital Garcia de Orta, Almada, PRT; 3 Urogynecology, Hospital Garcia de Orta, Almada, PRT

**Keywords:** abdominal pain in pregnancy, heavy vaginal bleeding, vaginal bleeding in pregnancy, abdominal pain in females, uterine hemorrhage, fallopian tubes, tubal pregnancy, interstitial pregnancy, ectopic pregnancy

## Abstract

An ectopic pregnancy is located outside the uterus, mostly found in the fallopian tubes. The main predisposing factors are usually related to anatomical and/or functional disturbances of the fallopian tubes. Typically, an ectopic pregnancy presents with vaginal bleeding or abdominal pain in the first trimester of pregnancy, although it may be asymptomatic. The diagnosis of ectopic pregnancy, which is based on transvaginal ultrasound associated with the serum beta fraction of human chorionic gonadotropin values, is of the highest relevance due to the mortality risk involved. We report the case of a 26-year-old woman who presented with a four-week history of amenorrhea and abdominal pain. Initially, the diagnostic hypothesis was a pelvic inflammatory disease, as the patient complained of pain during deep palpation of the lower quadrants of the abdomen, and gynecological observation detected malodorous discharge and cervical tenderness. Antibiotic treatment was initiated. The patient was later diagnosed with ectopic pregnancy, which was discovered during a routine pregnancy ultrasound. She was submitted to urgent laparotomy with intraoperative confirmation of the interstitial location of the gestational sac.

## Introduction

It is difficult to determine the exact incidence of ectopic pregnancies. Ninety-six percent of ectopic pregnancies are found in the fallopian tubes, including interstitial pregnancies (2%-4%), which occur specifically in the proximal portion of the tube. Most reports estimate an ectopic pregnancy rate of 6% to 16% among women who present to the emergency department with first-trimester bleeding and/or pain [1]. If the rupture occurs, being fallopian tubes highly vascularized, ectopic pregnancy results in catastrophic hemorrhage, accounting for 4% of pregnancy-related deaths, which constitutes an important cause of maternal mortality [1,2]. Considering the high mortality associated with this condition, early detection and treatment are of the utmost importance.

## Case presentation

A 26-year-old woman, with a history of four weeks of amenorrhea and a positive urine pregnancy test, presented to our Obstetric Emergency Department due to pelvic and lumbar pain. The patient reported having malodorous vaginal discharge that worsened during sexual intercourse and denied fever, constipation, or urinary symptoms. Relevant obstetric history included one induced abortion with curettage, two abortions without curettage, one cesarean section, and two vaginal deliveries. The patient was otherwise healthy and had no other complaints apart from rib pain that she attributed to an accidental fall months before.

The examination revealed malodorous yellow discharge with cervical tenderness, painful deep palpation, and decompression of both iliac fossae, with soft rebound tenderness. The patient complained of lumbar pain (positive renal Murphy sign), but it persisted with superficial palpation of the intercostal muscles, suggesting a previous thoracic lesion as a cause. An endovaginal ultrasound showed no gestational sac, without apparent pathological findings in both adnexal areas. The bloodwork showed no leukocytosis and a negative C-reactive protein. We explained to the patient the low probability of a visible gestational sac at such an early gestational age. The primary clinical hypothesis was pelvic inflammatory disease. The pain was relieved with acetaminophen therapy, and as the patient declined intramuscular ceftriaxone injection, a course of doxycycline and metronidazole was initiated.

After three weeks, with seven weeks and four days of amenorrhea, the patient returned to the Obstetric Emergency Department because of a possible ectopic pregnancy suspected on a routine outpatient obstetric ultrasound. The patient’s abdominal pain persisted without the addition of other new symptoms.

Laboratory values did not reveal any pathological finding, but a transvaginal ultrasound now detected a gestational sac and embryo in the uterine right horn, with cardiac activity.

**Figure 1:**
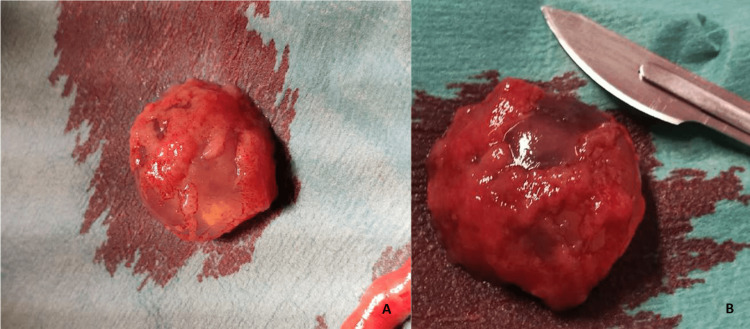
Interstitial ectopic pregnancy after surgery: (A) intact gestational sac; (B) gestational sac dimension compared to a scalpel.

The patient was immediately admitted to our department, and an open laparotomy was performed, with visual confirmation of an interstitial ectopic pregnancy on the right side of the uterus. The laparotomy approach was selected based on the experience of the medical team. We promptly performed a complete excision of the gestational sac, followed by right salpingectomy due to difficult hemostasis (Figure 1). Pathologic examination confirmed ectopic pregnancy inside the fallopian tube. The postoperative period was uneventful. The patient signed the informed consent for the publication of her clinical case before discharge.

We scheduled a follow-up appointment, and the patient was clinically asymptomatic, without complications related to the procedure. Clinical records show that this patient had already had an uneventful pregnancy after this episode, with the delivery of a healthy newborn at 39 weeks of gestation.

## Discussion

An ectopic pregnancy is an extrauterine pregnancy, most frequently localized in the fallopian tubes (96%). Of these tubal ectopic pregnancies, 2% to 4% are so-called interstitial pregnancies [1,3]. An interstitial pregnancy occurs when a gestational sac implants in the proximal portion of the fallopian tube, named the interstitial portion, which is 0.7 mm wide and 1 to 2 cm long and is surrounded by a muscular uterine wall [3]. It appears as a gestational swelling lateral to the round ligament. The myometrial layer surrounding the tube allows the gestation to expand and relatively protects it from rupture until seven to 16 weeks of gestation [4]. In interstitial pregnancies, a rupture could happen at any time in early pregnancy, although it occurs in 20% of cases progressing beyond 12 weeks of amenorrhea [5]. If the rupture occurs, the result is catastrophic hemorrhage, as this is a highly vascularized area. The maternal mortality rate of interstitial pregnancies is 2% to 2.5% [1,3].

The predisposing factors for interstitial pregnancy are generally similar to those for ectopic pregnancy and are typically conditions or events affecting the anatomy and/or function of the fallopian tubes, such as pelvic inflammatory disease, previous pelvic surgery, previous ectopic pregnancy, tumors, uterine anomalies, and pregnancy following assisted reproductive techniques. Ipsilateral salpingectomy is a risk factor that is exclusive to interstitial pregnancy [1,4,6].

Interstitial pregnancy typically presents with vaginal bleeding or abdominal pain in the first trimester of pregnancy, although it may also be asymptomatic [2].

This pathology presents a diagnostic challenge both clinically and radiologically. The diagnosis is based on transvaginal ultrasound associated with serum human chorionic gonadotropin (B-hCG) determinations. Interstitial pregnancy is suspected when ultrasonography demonstrates eccentric implantation of the gestational sac at the superior fundal level of the uterus. There are three sonographic criteria for diagnosing this entity: an empty uterine cavity, a chorionic sac seen separately and >1 cm from the most lateral edge of the uterine cavity, and a thin myometrial layer surrounding the chorionic sac [4].

Management of ectopic pregnancy includes either medical treatment (i.e., using folic acid antagonist methotrexate) or surgical treatment (i.e., conservative laparoscopy or laparotomy). Ultrasound and fast serum B-hCG level assessments, together with a high index of suspicion, facilitate early diagnosis and increase the success of conservative management of interstitial ectopic pregnancies. In the aforementioned medical approach, the morbidity from surgery and general anesthesia is eliminated, and there is potentially less tubal damage, cost, and need for hospitalization. The overall success rate of medical treatment in properly selected women is nearly 90%. International guidelines have established a scoring system used to determine eligibility for methotrexate therapy, which includes hemodynamic stability, no rupture, B-hCG < 5,000 IU/L, absence of cardiac activity, a gestational sac of <4 cm, absence of contraindications for methotrexate therapy, and patient compliance [3,5]. In terms of surgery, laparoscopy has become the recommended approach in most cases. Laparotomy is usually reserved for hemodynamically unstable patients or those with interstitial ectopic pregnancies; it also is a preferred method for surgeons inexperienced in laparoscopy and in patients in whom a laparoscopic approach is difficult (e.g., secondary to the presence of multiple dense adhesions, obesity, or massive hemoperitoneum).

## Conclusions

As interstitial ectopic pregnancy is a diagnostic and therapeutic challenge, the establishment and practice of a clinical approach with immediate diagnosis and appropriate therapeutic options are essential. The presented case report highlights an interstitial pregnancy diagnosed early in gestation. The identification and treatment of ectopic pregnancy are of extreme importance, given the high maternal risk and the nonviability of the pregnancy.
